# Impact of florfenicol dosing regimen on the phenotypic and genotypic resistance of enteric bacteria in steers

**DOI:** 10.1038/s41598-024-55591-8

**Published:** 2024-02-28

**Authors:** Jennifer Halleran, Hannah Sylvester, Megan Jacob, Benjamin Callahan, Ronald Baynes, Derek Foster

**Affiliations:** Department of Population Health and Pathobiology, Center of Veterinary Medicine, North Carolina State University, Raleigh, NC USA

**Keywords:** Microbiology, Molecular biology

## Abstract

The food animal sector’s use of antimicrobials is heavily critiqued for its role in allowing resistance to develop against critically important antimicrobials in human health. The WHO recommends using lower tier antimicrobials such as florfenicol for disease treatment. The primary objective of this study was to assess the differences in resistance profiles of enteric microbes following administration of florfenicol to steers using both FDA-approved dosing regimens and two different detection methods. Our hypothesis was that we would identify an increased prevalence of resistance in the steers administered the repeated, lower dose of florfenicol; additionally, we hypothesized resistance profiles would be similar between both detection methods. Twelve steers were administered either two intramuscular (20 mg/kg q 48 h; n = 6) or a single subcutaneous dose (40 mg/kg, n = 6). Fecal samples were collected for 38 days, and *E. coli* and *Enterococcus* were isolated and tested for resistance. Fecal samples were submitted for metagenomic sequencing analysis. Metagenomics revealed genes conferring resistance to aminoglycosides as the most abundant drug class. Most multidrug resistance genes contained phenicols. The genotypic and phenotypic patterns of resistance were not similar between drug classes. Observed increases in resistant isolates and relative abundance of resistance genes peaked after drug administration and returned to baseline by the end of the sampling period. The use of a “lower tier” antimicrobial, such as florfenicol, may cause an increased amount of resistance to critically important antimicrobials for a brief period, but these changes largely resolve by the end of the drug withdrawal period.

## Introduction

Antimicrobial resistance (AMR) is a national and global threat. In 2019, the United States reported that more than 2.8 million AMR infections and more than 35,000 human mortalities occur annually^1^. In the European Union (EU), AMR infections accounted for at least 25,000 deaths per year^2^. The cost of AMR infections is also significant, with estimates in the EU to be around $1.5 billion annually^2^. Increased antibiotic use in both the human and veterinary sectors has been correlated with an increased prevalence of AMR. While the sources of development and dissemination of antimicrobial resistance genes (ARGs) are unclear, use of antimicrobials in food producing species may contribute to the rise of AMR bacteria in humans^2^. The World Health Organization (WHO), the American Veterinary Medical Association and the American Association of Bovine Practitioners have each put forth recommendations regarding judicious antimicrobial use in food producing species^3–5^. The WHO has classified the different antibiotic classes into three main categories based upon their use in human medicine and any known human or animal origin resistance^3^. The three classifications include critically important, highly important and important antimicrobials. The WHO recommends the use of a “lower tier antimicrobial” (a highly important antimicrobial) for therapeutic treatment in food producing species^3^. This is intended to decrease the development and dissemination of ARGs to critically important antimicrobials used in humans.

One such “lower tier” antimicrobial commonly used in veterinary medicine is florfenicol, a fluorinated analog of thiamphenicol and chloramphenicol^6^. Florfenicol has an FDA-approved label for the treatment of bovine respiratory disease and foot rot at two different labeled dosing regimens, a lower intramuscular dose administered 48 h apart (20 mg/kg) for two doses, or a higher single, subcutaneous dose (40 mg/kg). Each of the two labeled dosing regimens has their own respective meat withdrawal interval, 28 days following the second intramuscular injection and 38 days after the subcutaneous injection. It has been documented that sub-therapeutic drug concentrations promote the proliferation of a resistant sub-population of bacteria^7^. Because of this phenomenon, administration of higher concentrations of an antibiotic early in the disease course should decrease the proliferation of a resistant sub-population of bacteria^7^. Both these florfenicol dosing regimens are administered parenterally, and previous work had demonstrated that low drug concentrations persist for long periods of time in the gastrointestinal tract, potentially exerting selection pressure on enteric bacteria^8^. Although resistance to florfenicol or chloramphenicol is not a major concern in human health, florfenicol administration has been associated with cross resistance against critically important antibiotics. In *E. coli*, common resistance patterns include resistance to florfenicol, ampicillin, ceftiofur and tetracycline^9^.

Antimicrobial resistance has been characterized through phenotypic or genotypic methods. Phenotypic methods assess bacterial growth after subjecting the bacterium to a particular concentration of an antimicrobial. Gram negative and Gram positive indicator organisms, commonly *E. coli* and *Enterococcus*, are used nationally for monitoring and surveillance of resistance in the food supply 10. Genotypic methods involve the identification of ARGs. It has been demonstrated that genotypic methods, such as whole genome sequencing can be utilized to predict phenotypic resistance^11^. High throughput sequencing techniques enable analysis of ARGs without culturing, potentially providing a broader assessment of AMR across a microbiome and not in specific indicator organisms. The main objective of this study was to assess resistance profiles following administration of florfenicol to steers for both FDA-approved dosing regimens with two different detection methods. The overall hypothesis for this study was that we would identify an increased prevalence of phenotypic resistance and ARGs in the steers administered the repeated, lower dose of florfenicol. A secondary hypothesis for this study was that the resistance patterns between both detection methods would be similar.

## Results

All twelve steers did well clinically throughout the study, no adverse reactions were noted. Feces were collected on all steers at all time points (n = 72 samples, 0 h, 72 h, 96 h, 168 h, 672 h, 912 h). Fecal enumeration of both *E. coli* and *Enterococcus* was conducted on selective media for all time points. Suspected *E. coli* colonies were confirmed positive by Indole testing (Thermo Scientific, Waltham, MA). *Enterococcus* were confirmed and speciated by MALDI-TOF. The four species identified were *Enterococcus casseliflavus*, *E. gallinarum*, *E. hirae*, and *E.mundtii* (Supplemental Table 1). *E. casseliflavus* (n = 142), *E. hirae* (n = 136)*,* and *E. mundtii* (n = 136) were the species most observed throughout the study period; *E. gallinaurum* was only identified twice, once at the start and once at the end of the study period.

### MIC values for *E. coli* and *Enterococcus*

The minimum inhibitory concentration (MIC) distribution, including median, inter-quartile range and range, were assessed for representative *E. coli* and *Enterococcus* isolates (Supplemental Tables 2 and 3). In this study, a total of 360 *E. coli* isolates and 299 *Enterococcus* isolates were used. At middle time points (72–168 h), the MIC value increased in *E. coli* against both ampicillin and tetracycline (Fig. 3F,G and Supplemental Fig. 4D,E). The MIC value then returned to baseline at the end of the study period; this time course change was not observed for any drugs in *Enterococcus*. To determine if there was a statistically significant difference in the MIC value for the organism and antibiotic pairing, predetermined individual Wilcoxon ranked sum tests were conducted with Bonferroni correction (p < 0.0125 would be significant). The four predetermined tests were as follows: in the high dose group, day 0 was compared to day 38; in the low group, day 0 was compared to day 28; at day 28, the low and high dose group MIC values were compared; at day 38, the low and high dose MIC values were compared. For *E. coli*, there were no significant differences in MIC found when analyzing ampicillin, cefazolin, minocycline or tetracycline at any of the predetermined time points; although numerous antimicrobials are tested on the NARMS plates, these were the only *E. coli* drug pairings that yielded growth. When comparing the MIC values for *Enterococcus* at the predetermined time points, there was a significant difference noted for gentamicin when comparing the low vs high dose group at day 28 (p = 0.004), but at day 38, the MIC value normalized in the high dose group (the withdrawal interval for the high dose group).

### Metagenomic sequencing analysis

The raw sequencing files underwent quality control, removal of bovine DNA and alignment against the Resistance Gene Identifier (RGI) within the Comprehensive Antimicrobial Resistance Database (CARD). At least 10 reads of a particular ARGs was needed to include the ARG in the dataset. It was then normalized to account for differences in the length of the ARG and the bacterial load in the samples. In addition, the data was normalized against the length of the 16S rRNA sequence. The normalization was done to avoid bias with the different lengths of ARGs and the bacterial burden. The outcome value refers to the normalized AMR gene abundance. As demonstrated in Table 1, the range in ARGs detected was from 232 to 273, with the largest number of ARGs detected at time point 72 h; this would have occurred 24 h after the second low dose of florfenicol was administered, so it is not surprising to have an increase in ARGs present. Time point 672 h (28 days) had the lowest amount of ARGs; twenty eight days is the FDA approved meat withdrawal interval for the low dose (20 mg/kg IM) of florfenicol. There was an overall rise in ARGs present at the end of the study period (240 ARGs, time point 912 h, 38 days) when compared to time point 672 (28 days).

**Table 1:** This table demonstrates the averaged mapped reads in CARD, the average number of 16S alignments and the average number of ARG terms aligned for each time point.

	Average mapped reads against CARD	Average 16S alignment	Average ARG
Time 0	51,077	19,876	271
Time 72	66,466	19,267	273
Time 96	79,321	17,817	270
Time 168	107,164	20,438	269
Time 672	39,310	14,705	232
Time 912	47,675	17,884	240

Numbers listed include data from both dosing groups.

**Figure 1:**
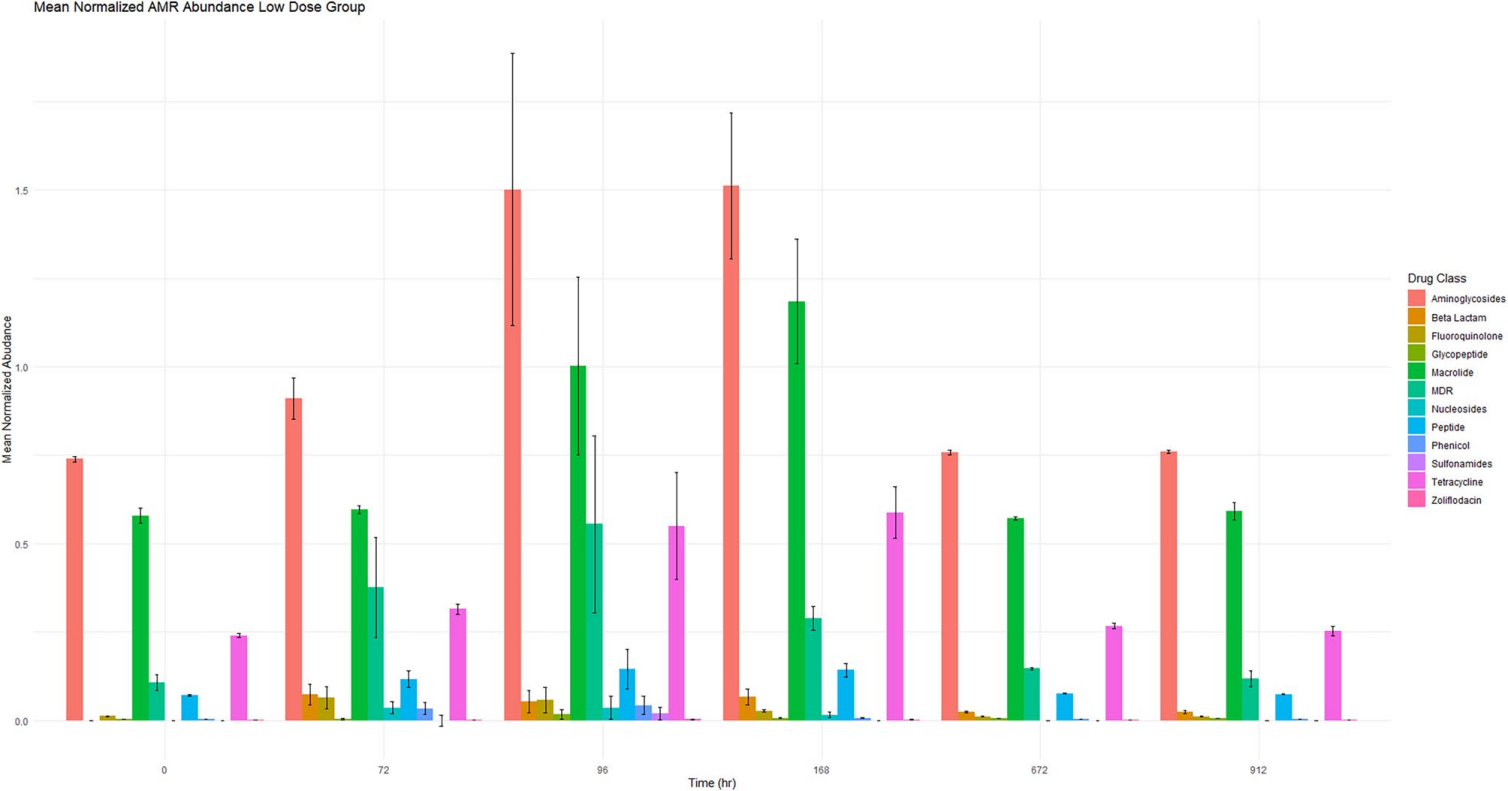
This figure demonstrates the mean normalized AMR gene abundance for the low dose treatment group per drug class at each time point. The normalized mean abundance with standard error is shown.

**Figure 2:**
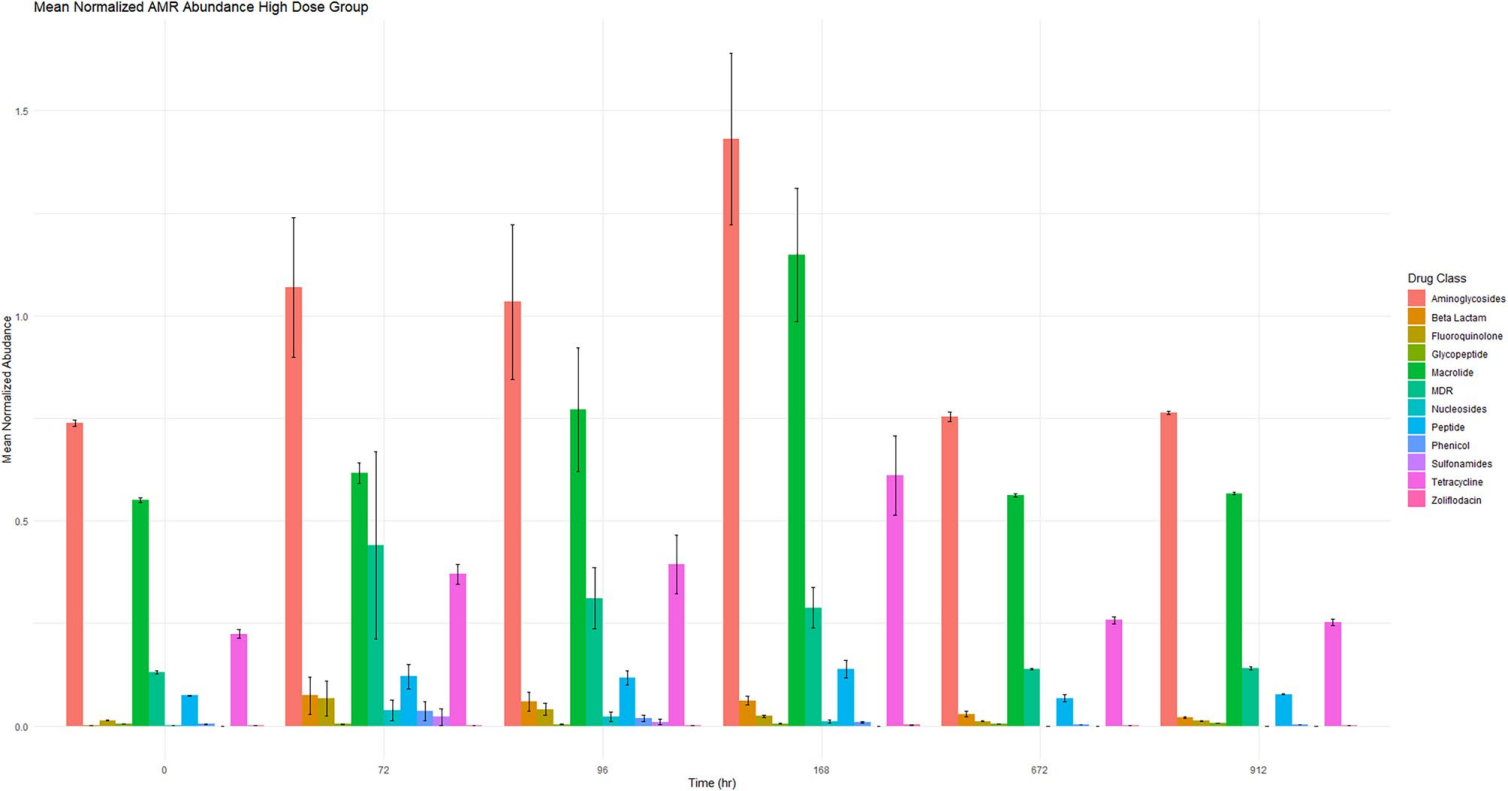
This figure demonstrates the mean normalized AMR gene abundance for the high dose treatment group per drug class at each time point. The normalized mean abundance with standard error is shown.

Figures 1 and 2 show the mean normalized AMR gene abundance for each drug class for the low (Fig. 1) and high dose (Fig. 2) group respectively (mean with standard error). In the low and high dose group, aminoglycosides are the most abundant resistance determinant. Following aminoglycoside ARGs, macrolide ARGs, tetracycline ARGS and ARGs that confer resistance to multiple classes (MDR) are the most abundant. To determine if there was a statistically significant difference between the mean normalized AMR abundance between dosing groups, a Wilcoxon ranked sum test with Bonferroni correction at predetermined time points was conducted. The only statistically significant comparison was time point 0 h to 912 h in the low dose group, and then again in the high dose group for beta lactams, there was a statistically significant difference with 912 h having a higher mean normalized abundance (both groups, n = 6 steers per group per time point, both comparisons, p = 0.004). Although the mean normalized AMR abundance may be increased, at this time, it is not fully understood what effect this value has on the dissemination of ARGs in the food supply.

### Comparison of phenotypic and genotypic resistance detection methods

**Figure 3:**
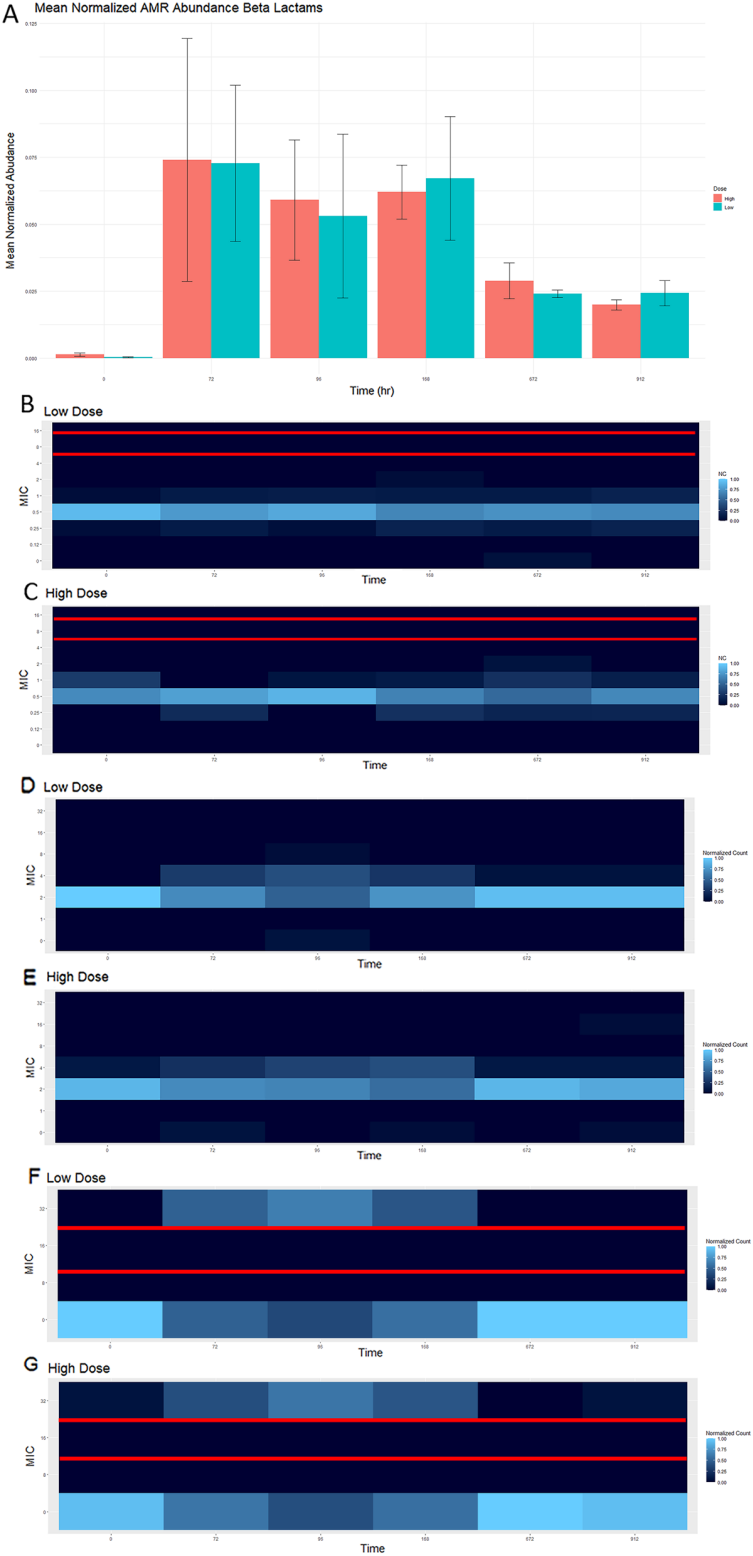
This figure is demonstrating the genotypic beta lactam (**A**) and phenotypic resistance of Enterococcus to ampicillin (**B**, **C**) and *E. coli* to both cefazolin (**D**, **E**) and ampicillin (**F**, **G**). The genotypic data was obtained through comparison against CARD. Normalized mean abundance with standard error is shown (**A**). Phenotypic resistance patterns were obtained through inoculation of gram positive and gram negative NARMS plates. The normalized isolate count (NC) is shown for each figure. For Enterococcus, resistance occurs with an MIC ≥ 16 and is susceptible with an MIC value ≤ 8 (**B**, **C**). For *E. coli*, resistance to cefazolin occurs with an MIC value of ≥ 16 and is susceptible with an MIC value ≤ 2 (**D**, **E**). For *E. coli*, resistance to ampicillin occurs with an MIC value of ≥ 32 and is susceptible with an MIC value ≤ 8 (**F**, **G**).

**Figure 4:**
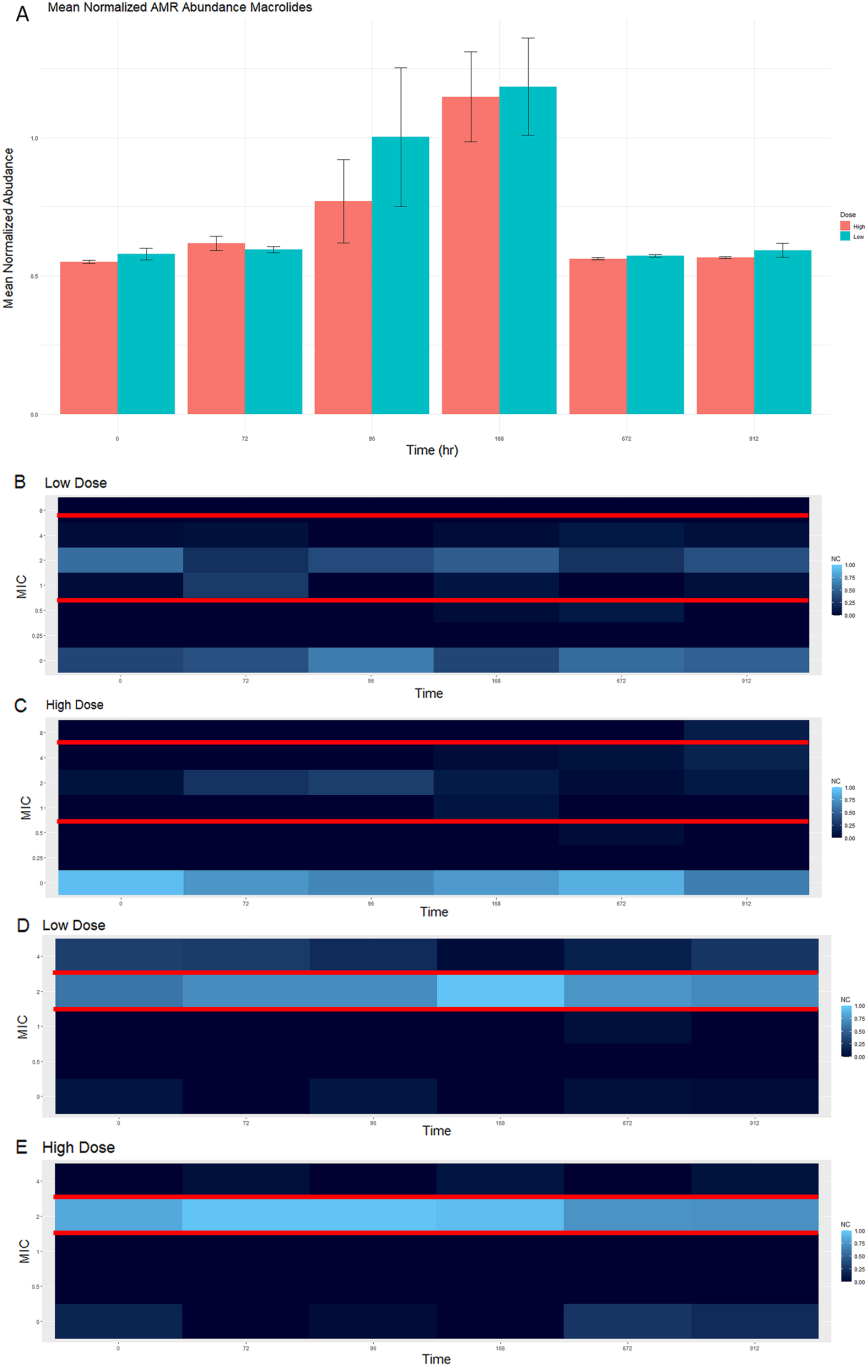
This figure demonstrates the genotypic macrolide (**A**) and phenotypic resistance of Enterococcus to erythromycin (**B**, **C**) and quinupristin (**D**, **E**). The genotypic data was obtained through comparison against CARD. Normalized mean abundance with standard error is shown (**A**). Phenotypic resistance patterns were obtained through inoculation of gram positive NARMS plates. The normalized isolate count (NC) is shown for each figure. For Enterococcus, resistance to erythromycin occurs with an MIC ≥ 8 and is susceptible with an MIC value ≤ 0.5 (**B**, **C**). For Enterococcus, resistance to quinupristin occurs with an MIC value of ≥ 4 and is susceptible with an MIC value ≤ 1 (**D**, **E**).

**Figure 5:**
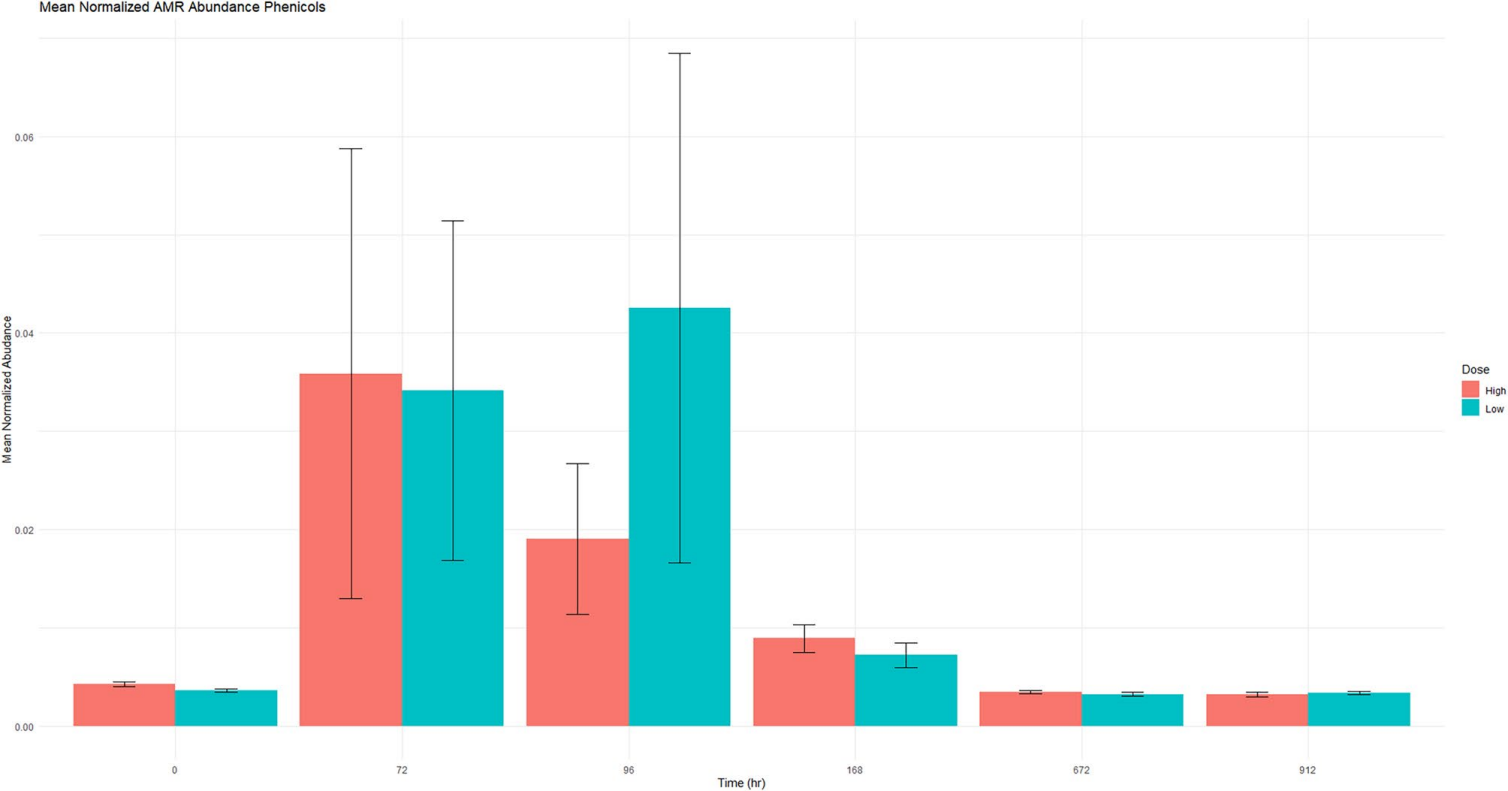
This figure demonstrates the genotypic phenicol resistance. The genotypic data was obtained through comparison against CARD. Normalized mean abundance with standard error is shown.

**Figure 6:**
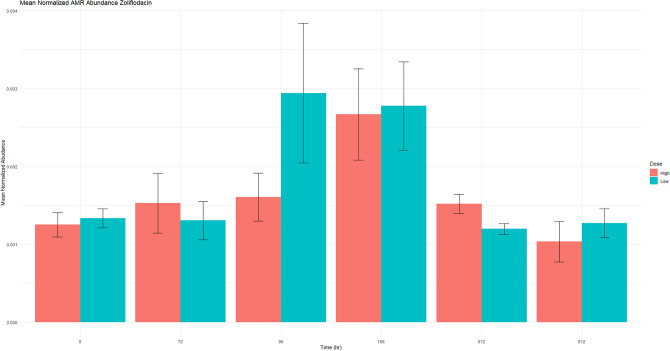
This figure demonstrates the genotypic resistance against zoliflodacin, a new medication currently in research trials for treatment of gonorrhea. Normalized mean abundance with standard error is shown.

**Figure 7:**
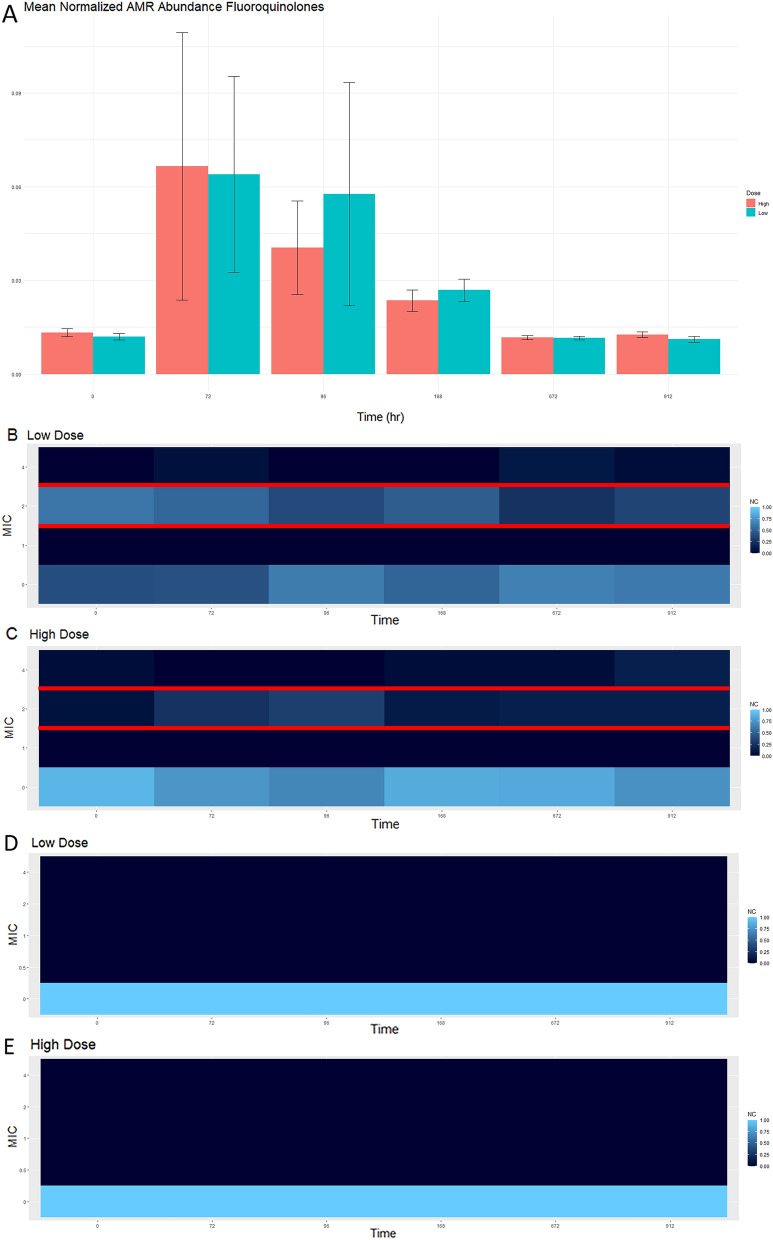
This figure demonstrates the genotypic fluoroquinolone (**A**) and phenotypic resistance of *Enterococcus* (**B**, **C**) and *E. coli* (**D**, **E**) to ciprofloxacin. The genotypic data was obtained through comparison against CARD. Normalized mean abundance with standard error is shown (**A**). Phenotypic resistance patterns were obtained through inoculation of gram positive and gram negative NARMS plates. The normalized isolate count (NC) is shown for each figure. For Enterococcus, resistance occurs with an MIC ≥ 4 and is susceptible with an MIC value ≤ 1 (**B**, **C**). For *E. coli*, resistance occurs with an MIC value of ≥ 0.12 and is susceptible with an MIC value ≤ 0.06 (**D**, **E**).

Each drug class was examined in more detail with comparisons made between genotypic and phenotypic resistance patterns found for *E. coli* and *Enterococcus*. The hypothesis for this study was twofold; first, there would be an increased abundance of phenotypic resistance and ARGs in the low dose group. Second, the resistance pattern would be similar regardless of detection method. In general, there was an increase in mean normalized AMR abundance seen 72 h following administration of florfenicol, with a return to baseline (time point 0) at the end of the study period in both dosing groups (Figs. 3A, 4, 5, 6, 7A). There was no statistically significant difference noted when comparing mean normalized AMR abundance between dosing groups at any of the predetermined time points. When comparing the phenotypic resistance data (MIC values from NARMS plates), only one antimicrobial class (gentamicin) had a significant difference in MIC values between dosing groups at time point 672 h, but this was not found at the end of the study period.

Phenotypically, the general trend of an increasing MIC value after florfenicol administration with return to baseline was only observed for certain pairings. This was observed with *E. coli* and both cefazolin (Fig. 3D,E) and ampicillin (Fig. 3F,G) and was similar to the genotypic resistance pattern of beta-lactam ARGs. When phenotypically assessing *Enterococcus* and ampicillin, the same trend was not present (Fig. 3B,C). *Enterococcus* MIC to ciprofloxacin in the high dose group (Fig. 7C) followed the same trend as the ARGs (Fig. 7A). Increased MIC values in *Enterococcus* to quinupristin were observed throughout the study period Fig. 4D); however, the final day of the study and time point 0 are similar, indicating the administration of florfenicol did not worsen the phenotypic resistance of *Enterococcus* and quinupristin. ARGs against zoliflodacin, a new antimicrobial for treatment of gonorrhea was found in this study, but at low abundance levels (Fig. 6). Plots for aminoglycosides, glycopeptides, peptides, tetracyclines and can all be found in the supplemental material (Figs. S1–S4). In summary, similar resistance patterns with phenotypic versus genotypic methods were observed for only select antimicrobial-bacteria pairings.

**Figure 8:**
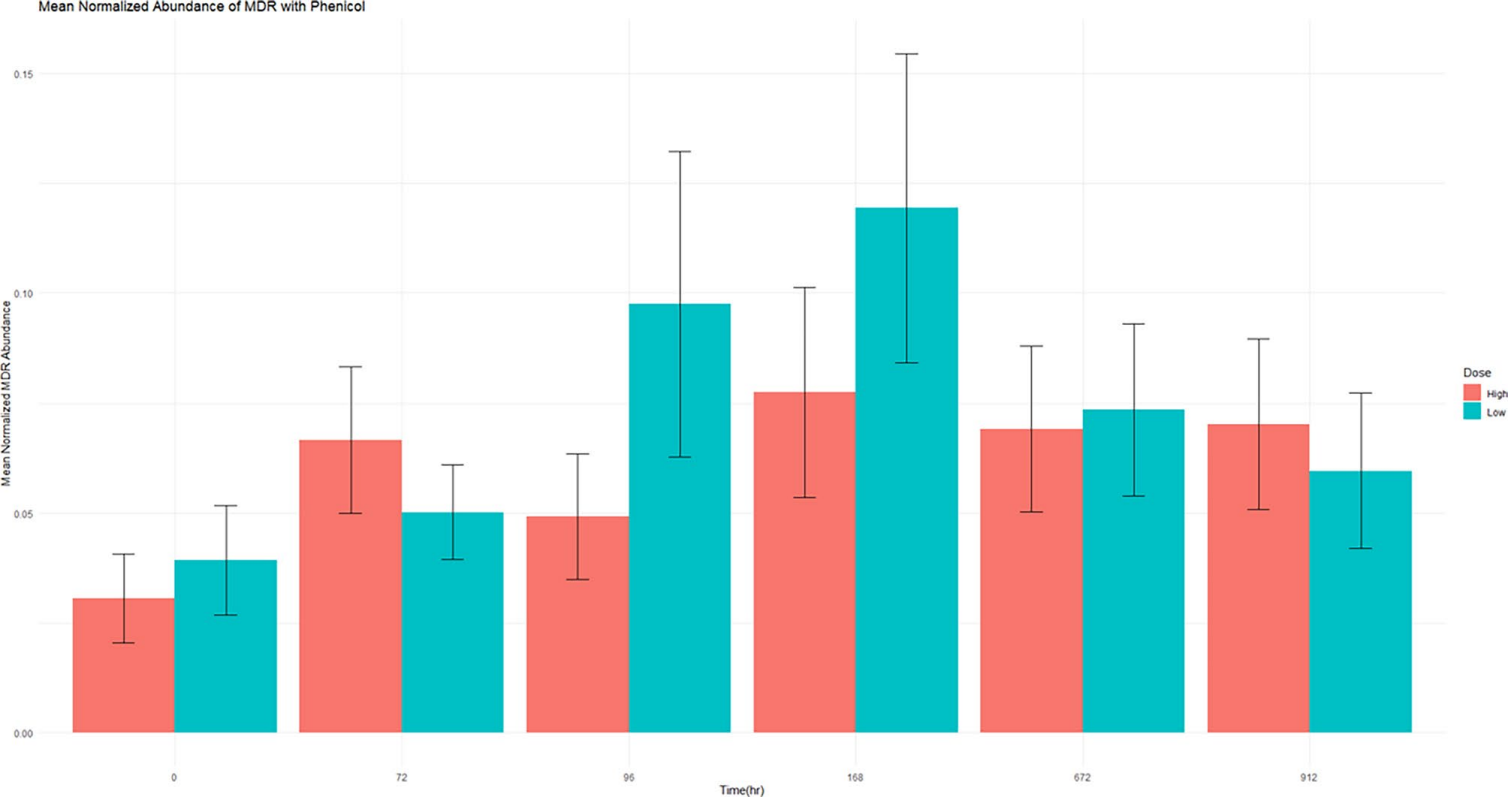
This figure demonstrates the genotypic resistance of MDR patterns that contain phenicol. Normalized mean abundance with standard error is shown.

Although resistance to phenicol is not a concern in human medicine, it is interesting to observe the resistance pattern against the phenicol class, as this is the class administered in this study. The mean normalized ARG abundance did increase in both dosing groups following administration, but it was short lived and quickly returned to baseline (Fig. 5). Beta lactams (Fig. 3), macrolides (Fig. 4) and fluoroquinolones (Fig. 7) are all classes of antimicrobials of critical importance in human medicine. In general, there was increase in mean normalized ARGs for all three, but by the end of the study period, the abundance returned to baseline; this could potentially indicate with florfenicol administration, there is not an increased probability of ARGs being transmitted in the food supply at the time of slaughter. Multidrug resistance in the resistome was defined as an ARG with that could potentially confer resistance against 3 or more different antimicrobial classes. In this data set, 23 ARGs were classified as MDR. Two genes acted by target protection, one altered the antibiotic drug target, and the remaining 20 genes were efflux pumps. Ten of the 23 identified MDR genes contained phenicol resistance mechanisms. The presence of MDR genes with phenicol increased in the middle of the study period, but no statistically significant difference detected between time 0 and the end of the sampling period (Fig. 8).

## Discussion

The main objective of this study was to assess the resistance profiles following administration of florfenicol to steers for each of the FDA-approved dosing regimens for cattle using two detection methods. Our overall hypothesis for this study was there would be an increased prevalence of phenotypic resistance and ARGs in the steers administered the repeated, lower dose of florfenicol, with a secondary hypothesis of similar resistance patterns found using both phenotypic and genotypic methods. In summary, resistant MIC values were observed in both dosing groups, but returned to baseline at the end of the study period. Genotypically, numerous ARGs were detected, but the mean normalized AMR abundance did not significantly differ between dosing groups. A general trend can be observed in the ARGs data, where an increase in abundance is present after florfenicol administration with a return to baseline. Phenotypically, this pattern was mimicked only in ampicillin and cefazolin MIC values for *E. coli* and in ciprofloxacin MIC values for *Enterococcus*. Ten identified MDR genes contained phenicol resistance mechanisms.

Both *florR* and *fexA* are common plasmids and chromosome encoded exporters associated with florfenicol resistance. The *floR* is a plasmid or chromosome encoded phenicol exporter, that has been found in *E. coli* from cattle, swine and poultry in *Klebsiella pneumonia* and *Vibrio cholera* isolates^12–14^. Some plasmids containing the *floR* gene are multidrug resistance plasmids^13^. In *Salmonella enterica*, *floR* is found on a chromosome located next to a tetracycline resistance operon; this would convey cross resistance and has been found in different animal species, suggesting mobility^12^. *fexA* is a plasmid encoding chloramphenicol exporter^16^. The *fexA* gene encodes for efflux pumps within major facilitator superfamily, which has been shown to be part of a transposon^15^. Pumps associated with the major facilitator superfamily are dominant in *E. coli* and have been shown to transport many antibiotic compounds. Therefore, these pumps can confer cross resistance to macrolides, fluroquinolones, tetracyclines, trimethoprim and chloramphenicol^16^. Both *florR* and *fexA* can confer resistance to multiple drug classes.

In this study, an MDR gene was defined as one that could confer resistance to 3 or more antimicrobial classes. Of the MDR genes, phenicol resistance was associated with 10. While resistance to florfenicol or chloramphenicol is not a human health concern, co-resistance with florfenicol has been associated with antimicrobial classes that are considered critically important antimicrobials in human medicine, including beta lactams. Common resistance patterns observed with florfenicol include resistance to ampicillin, ceftiofur and tetracycline^9^. In this study, phenicol resistance alone was associated with mutations in the 23 s rRNA to allow for target alteration. Phenicol resistance contained in MDR genes demonstrated resistance mechanisms associated with antibiotic alteration, protection, and drug efflux pumps mechanisms. Efflux pumps (*florR*, *fexA/fexB*, *pexA/pexB*), rRNA methyltransferase (*cfr*), chloramphenicol acyltransferases (*catA*, *catC*) and ribosomal protection proteins (*optrA*, *poxtA*) are MDR mechanisms that have been characterized to convey phenicol resistance^15,17–21^. All of the above listed MDR genes were identified in this study at time points 0, 72, 96 and 168 h, but they were not identified at time points 672 or 912. At these last two time points, the common gene family for conveying MDR resistance (containing phenicol) was a point mutation in the 23 s rRNA subunit that allowed for resistance and an ABC-F ATP-binding cassette protein superfamily. This ABC-F gene is unlike other ABC proteins as resistance is associated with ribosomal protection and not efflux. These gene families may be a concern where the mechanism of action of the antimicrobial is the ribosome (macrolides).

Most published studies assess the relationship between phenotypic resistance and genotypic resistance, with bacterial isolates undergoing whole genome sequencing or specific PCR gene identification. Phenotypic methods have been used in the past to assess AMR, with in vitro antimicrobial susceptibility testing (AST) as the gold standard and the main detection method studied correlating clinical outcomes^22^. Genotypic methods have now become popular for surveillance and new resistance gene discovery. Each method provides different insight on assessing AMR and different results may be generated. Therefore, perhaps it is best to use them in conjunction with one another. In this study, to determine the phenotypic resistance profile, human specific Sensititre plates were utilized; this would allow for observations to be made regarding the public health impact of florfenicol administration in cattle. These plates were decided upon over typical Sensititre surveillance plates because of the increased breadth of antimicrobial classes tested. In this study, only certain phenotypic resistance patterns correlated with the genotypic data. One reason may be only *E. coli* and *Enterococcus* were evaluated. Both organisms have historically been used as indicators and are utilized to document prevalence and emergence of resistance in many surveillance settings^10^. In the genotypic dataset, all bacterial organisms that could be present in the sample were assessed for ARGs. Different bacterial organisms, aside from *E. coli* and *Enterococcus*, may harbor the respective ARGs. The use of longer read sequencing would need to be utilized to fully understand which bacterial agent is harboring which ARGs. Alternatively, *E. coli* and *Enterococcus* may have the ARGs, but they were not being expressed. In addition, the relative abundance of *E. coli* and *Enterococcus* may have been low in the gastrointestinal tract of ruminants, suggesting sequencing may not have been sensitive enough to detect ARGs associated with the resistance profile observed phenotypically. It does support the need for both modalities to fully characterize resistance present, especially when one considers the level of sensitivity desired for detecting AMR, or studying different, more prevalent indicator organisms. If a more sensitive detection method is needed, then genotypic assessment may be warranted.

There are several limitations to this current study. There was no control group (no florfenicol administration) in this study. While it would be ideal to have a control group to observe phenotypic and genotypic changes in the face of no antimicrobials, this was not the primary goal of the study. Resistance patterns at time point 0, prior to any florfenicol administration, were used a baseline for comparison to other predetermined time points throughout the study period. First, for a true comparison of phenotypic and genotypic resistance patterns, either multiple species of bacteria should have been evaluated, or a different sequencing modality should have been employed. Future work may include the use of longer read sequencing modalities to identify which bacterial agent is responsible for the ARGs documented. This would be important in identifying bacterial species that would potentially be better indicator organisms for AMR transmission potential. A second limitation, which will act as a scaffold for future work, is understanding the significance of a normalized abundance value. The values obtained in this study may or may not be significant in terms of horizontal gene transfer and AMR expression. More work needs to be performed to fully understand how these normalized abundances will contribute to the dissemination of AMR through communities.

In this study, resistance was identified in both dosing group with both detection methods. Phenicol resistance appears to be associated with co-resistance to multiple antibiotic classes in both dosing groups, but the significance in the dissemination to the food supply and human health is unknown. Regardless of dosing group, the use of florfenicol as a “lower tier” antimicrobial may not reduce the overall development, persistence and potential transmission of ARGs relative to other “higher tier” drug classes, but this resistance largely returns to baseline by the end of the drug withdrawal time. Future work needs to be performed to assess the effects of administering other higher and lower tier drug classes of in steers on phenotypic and genotypic resistance patterns.

## Materials and methods

### Animals and treatment

This study was approved by North Carolina State University’s Intuitional Animal Care and Use Committee. All methods and animal work were carried in accordance with animal welfare guidelines and with the ARRIVE guidelines. Twelve healthy 6–7 month old steers (153.3–251.8 kg) were enrolled in the study. The steer study size was based on previous gastrointestinal pharmacokinetic studies to demonstrate differences between the two dosing regimens^8,23,24^. They were judged healthy by a physical exam on presentation and had no previous documentation of any antimicrobial administration. After a 3-day period of acclimation, the steers underwent gastrointestinal surgery for a different study^8^. At the time of surgery, steers received either intravenous flunixin meglumine (2 mg/kg, Banamine®, Merck Animal Health) or transdermal flunixin meglumine (3.3 mg/kg, Banamine® Transdermal, Merck Animal Health).

Twenty-four to 48 h after surgery, the steers were dosed with either 20 mg/kg florfenicol (Nuflor®, Merck Animal Health) intramuscularly every 48 h (n = 6) twice, or a single 40 mg/kg subcutaneous dose (n = 6). The steers were randomly assigned via number generator to either of the treatment groups. The steers were housed in pairs (one from each treatment group) and fed grass hay with supplemental grain and free access to water for the duration of the study.

### Collection of feces

Feces were collected manually from the rectum. Time points for feces collection were 0, 72 h, 96 h, 168 h, 672 h (28 days) and 912 h (38 days). The samples were placed into bags (Whirlpak®, Nasco, Fort Atkinson, WI) and stored on ice until microbiological analysis. Prior to microbiological analysis, 6 aliquots of feces were placed in cryovials and stored at – 80 °C for future use.

### Isolation of *E. coli* and *Enterococcus* from feces

The time points studied were as follows: 0 h, 72 h, 96 h, 168 h, 672 h, and 912 h. One gram of feces was weighed and placed into both 9 mL of EC broth (Oxoid Ltd., Basingstoke, Hampshire, England) for *E. coli* isolation and 9 mL of Phosphate Buffered Saline (PBS, Fisher Bioreagents, Waltham, MA) for *Enterococcus* isolation. The samples were vortexed and subsequently serially diluted tenfold into sterile phosphate buffer. The diluted samples were plated in triplicate (100 µL) on selective media; *E. coli* dilutions were plated on MacConkey agar (Difco™ Becton, Dickinson and Company, Sparks, MD) and *Enterococcus* dilutions onto m Enterococcus agar (Difco™ Becton, Dickinson and Company, Sparks, MD). The MacConkey Agar plates were incubated overnight at 37 °C, while the *Enterococcus* plates were incubated for 48 h at 37 °C. Dilutions that yielded colony counts of 30–300 were counted and the three replicates averaged to determine the concentration of both *E. coli* and *Enterococcus* at each time point. From the plates that were used to determine the concentration of *E. coli* and *Enterococcus*, 8 isolates were randomly selected and streaked for isolation onto Columbia agar with 5% sheep blood (Remel, Lenexa, KS) and incubated overnight at 37 °C. After incubation, each suspected *E. coli* isolate was evaluated for indole production (Indole Reagent Kovacs, Remel, Lenexa, KS). Each isolate was then stored in a cryogenic vial containing LB Broth (Sigma-Aldrich, St. Louis, MO) supplemented with 25% glycerol (Fisher BioReagents™, Fisher Scientific, Waltham, MA). They were vortexed and frozen at − 80 °C as pure growth. *Enterococcus* isolates were speciated using MALDI-TOF mass spectrometry (Biomerieux).

### Antimicrobial susceptibility testing of *E. coli* and *Enterococcus*

To determine if there is a statistically significant difference in the phenotypic resistance pattern between the low and high dosing groups, it was determined that 62 bacterial isolates would be needed for each dosing group during the study period to yield a significance with a power of 80%^8^. This sample size was calculated based upon proportion of resistant isolates from previous work and performed in R using the power calculation tool for proportions. Frozen bacterial isolates were grown overnight on Columbia agar with 5% sheep blood prior to inoculation of microbroth dilution plates. Sensititre plates for both human gram negative (GN4F plate, Fisher, Waltham, MA) and gram positive (GPALL1F plate, Fisher, Waltham, MA) plates were utilized by following manufacturer directions. Briefly, the samples were standardized to 0.5 McFarland in demineralized water (Sensititre sterile water). After standardization, 10 uL of *E. coli* isolates and 30 uL of *Enterococcus* isolates were removed from the demineralized water and placed in Mueller Hinton broth (Sensititre Mueller–Hinton broth, Fisher, Waltham, MA). The newly inoculated Mueller Hinton Broth was vortexed, and using the Sensititre AIM automatic inoculation machine, 50 uL was inoculated within each well on the appropriate plate type. The plates were then sealed and placed in Sensititre ARIS 2 × for incubation and automatic reading. The gram negative plates were incubated for 18 h and then read by the computer and manually. The gram positive plates were incubated for 24 h and then read by the computer and manually.

For every fifth NARMs plate run, a purity plate was performed to ensure the entire system was clean and functional. The purity was inoculated from the positive control well, incubated at 37 °C overnight and assessed for growth by visual inspection.

### Statistical analysis of susceptibility outcomes

The MIC for each bacterial isolate at the specified time point for the dosing group was recorded for each drug on the respective plate. Predetermined individual Wilcoxon-Ranked Sum tests were conducted to assess MIC values for each organism. The four predetermined Wilcoxon Ranked Sum test are as follows: in the high dose group, time 0 h was compared to time 912 h in the low group, time 0 h was compared to time 672 h; at time 672 h, the low and high dose group MIC values were compared; at time 912 h, the low and high dose MIC values were compared. To account for multiple comparison, Bonferroni correction was utilized and a p value less than 0.0125 would be deemed statistically significant. To construct heat maps, the isolates per time point per dosing group were normalized by determining the percentage of total isolates at that time point that had each particular MIC value. Statistical analysis was conducted utilizing R Software, Version 3.6.3 “Holding the Windsock.” The bacterial isolates were classified as susceptible, intermediate and resistant based on human breakpoints as per the CLSI guidelines for *E. coli* and *Enterococcus* for the drugs present in the gram negative and gram-positive plates.

### Fecal DNA extraction

The same time points were studied for the genotypic portion. They are as follows: 0 h, 72 h, 96 h, 168 h, 672 h, and 912 h. Fecal DNA was extracted using ZymoBIOMICS DNA Miniprep Kit (Zymo Research, Catalog Number D4300, Irvine, CA). Briefly, the aliquoted fecal samples were thawed. The fecal samples were lysed and vortexed at the highest speed for 15 min. Then, after centrifugation (10,000×*g* for 1 min), the supernatant was added to a filter to clean the sample. A binding solution was added to enable DNA binding to a spin column filter, followed by subsequent washing. Finally, the DNA was eluted off the column with DNase/RNase free water. A prep solution was added to allow for subsequent sequencing analysis.

### DNA prep and metagenomic sequencing

Genomic DNA (gDNA) was submitted to the North Carolina State Genomic Sciences Laboratory (Raleigh, NC, USA) for Illumina NGS library construction and sequencing. Prior to library preparation, the isolated DNA template was quantified, and quality was assessed using an Agilent 2200 Tapestation High Molecular Weight DNA assay (Agilent Technologies, USA). Library construction was performed using an Illumina TruSeq Nano Library kit with provided protocol. Briefly, the gDNA was fragmented using a Covaris S220 Ultrasonicator (Covaris, USA) and purified using sample purification beads included in the TruSeq kit. The fragments were then end-repaired, followed by 350 bp insert size-selection using sequential bead isolation steps. After adapter ligation, the library was enriched by PCR amplification. The amplified library was checked for quality and final concentration using the Agilent 2200 Tapestation (D1000) before sequencing on an Illumina NovaSeq Sequencer utilizing a S4 150 × 2 PE flow cell (Illumina, USA). Raw base call file (bcl) were then de- multiplexed by sample into discrete .fastq files for data analysis.

### Metagenomic sequencing analysis

The raw sequencing files were analyzed utilizing North Carolina State University’s high processing computer. The sequences were first trimmed and underwent quality control filtering using fastp^25^. The minimum length was to set 36 nucleotides, base correction was established, the first and last four nucleotides from read 1 and read 2 were removed and the reads were merged into a single output file. Following trimming and quality filtering, reads had a mean length of 150 pairs before merging. Reads with Phred scores of 30 were utilized for downstream analysis. From there, bowtie2 was used to remove bovine DNA by aligning against the *Bos taurus* genome UMD 3.1.1^26,27^. Default parameters on bowtie2 were used. The files were then converted into .fastq files utilizing SAMtools^28^. Sequences were then aligned to the Resistance Gene Identifier (RGI) within the Comprehensive Antimicrobial Resistance Database (CARD)^29^. The metagenomic analysis pipeline was used in RGI for analysis. It is difficult to determine sample sizes with metagenomic sequencing technologies. While there is currently much research being conducted regarding this topic, it is not very well known yet the best way to conduct a power calculation to yield statistically significant sample sizes. Based upon an article by Li et al. with our primary objective in mind, the research team determined the best way to calculate the sample size would be based upon RNA sequencing count data and a Poisson distribution^30^. To do this, an RnaSeqSampleSize calculator was used^31^. Using this calculator, it was determined that 64 ARGs would be needed per treatment group to detect a statistically significant difference in ARGs between treatment groups. This is based upon an alpha of 0.125 (multiple comparisons), the number of references sequences in the CARD database (5192 sequences), expecting only 10 ARGs to be present and only needing 10 reads per ARG to be counted as present. Table 1 demonstrates the averaged mapped reads in CARD, the average number of 16S alignments and the average number of ARG terms aligned for each time point.

To analyze the ARG data, at least 10 reads were needed to be present per ARG to be counted present within the sample^32^. The reads were then organized by resistance to drug class. Multidrug resistance (MDR) was determined by resistance to at least 3 drug classes. The ARG data was then normalized to the microbial data obtained utilizing the following formula^33,34^:

$$ {\text{AMR Gene Abundance }} = \, \sum \frac{{{\text{N}}_{{{\text{AMR}} - {\text{Seq}}}} {\text{X L}}_{{{\text{reads}}}} /{\text{L}}_{{\text{AMR Ref}}} }}{{{\text{N}}_{{{\text{16Seq}}}} {\text{X L}}_{{{\text{reads}}}} /{\text{L}}_{{{\text{16seq}}}} }} $$

where N_AMR-Seq_ is the number of alignments to one specific AMR gene sequence, L_reads_ is the sequence length of our Illumina reads (150 bp), L_AMR-Ref_ is the sequencing length of the corresponding AMR gene in the CARD database, N_16Seq_ is the number of alignments to the 16S sequence and L_16seq_ is the average length of the 16S sequence (1550 bp)^35^. The number of alignments to the 16S sequence was determined using Metaxa2^36^.

For each steer, any present ARG pertaining to the same drug class was summed for a total normalized AMR gene abundance for each individual steer for each drug class. Then, for each dosing group (Low vs High), mean normalized ARG abundance was calculated with standard error for each drug class. These classes were then plotted using R and ggplot 2^37^. For statistical analysis, Wilcoxon ranked sum tests were performed at predetermined time points to assess the abundance of resistance to antibiotic drug class between dosing groups and at different time points. Within the low dose group, time points 0 and 28 days were compared and day 28 was compared to day 38. In the high dose group, time point 0 and 38 days were compared. At time point 38 days, the low dose and high dose group were compared. Bonferroni correction was utilized for the repeated Wilcoxon ranked sum testing, yielding a p value of less than 0.0125 to be statistically significant.

### Supplementary Information

Supplementary Figure S1.

Supplementary Figure S1.

Supplementary Figure S1.

Supplementary Figure S2.

Supplementary Figure S2.

Supplementary Figure S3.

Supplementary Figure S3.

Supplementary Figure S4.

Supplementary Figure S4.

Supplementary Figure S4.

Supplementary Figure S5.

Supplementary Figure S5.

Supplementary Table 1.

Supplementary Table 2.

Supplementary Table 3.

## Data Availability

All raw sequencing files are available at http://www.ncbi.nlm.nih.gov/bioproject/1065888. The submission number is SUB14126924 with a BioProject ID of PRJNA1065888.
