# Supplementary figures and images for: Impact of florfenicol dosing regimen on the phenotypic and genotypic resistance of enteric bacteria in steers
Source: Sci Rep. 2024 Feb 28;14:4920. doi: 10.1038/s41598-024-55591-8 (PMC10901817; doi:10.1038/s41598-024-55591-8)

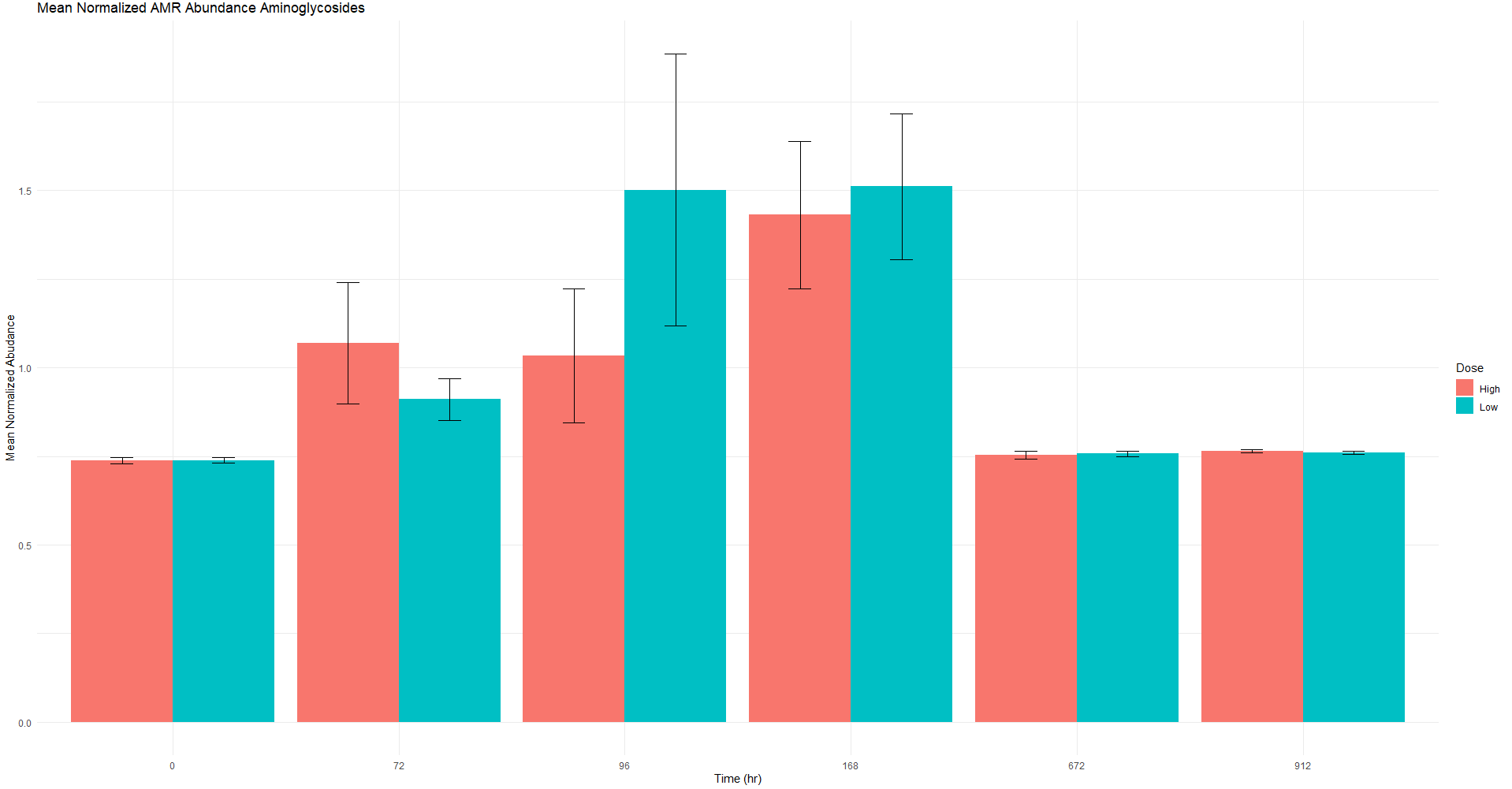

Supplement: Supplementary file 1 — Supplementary Figure S1. [file 41598_2024_55591_MOESM1_ESM.png]

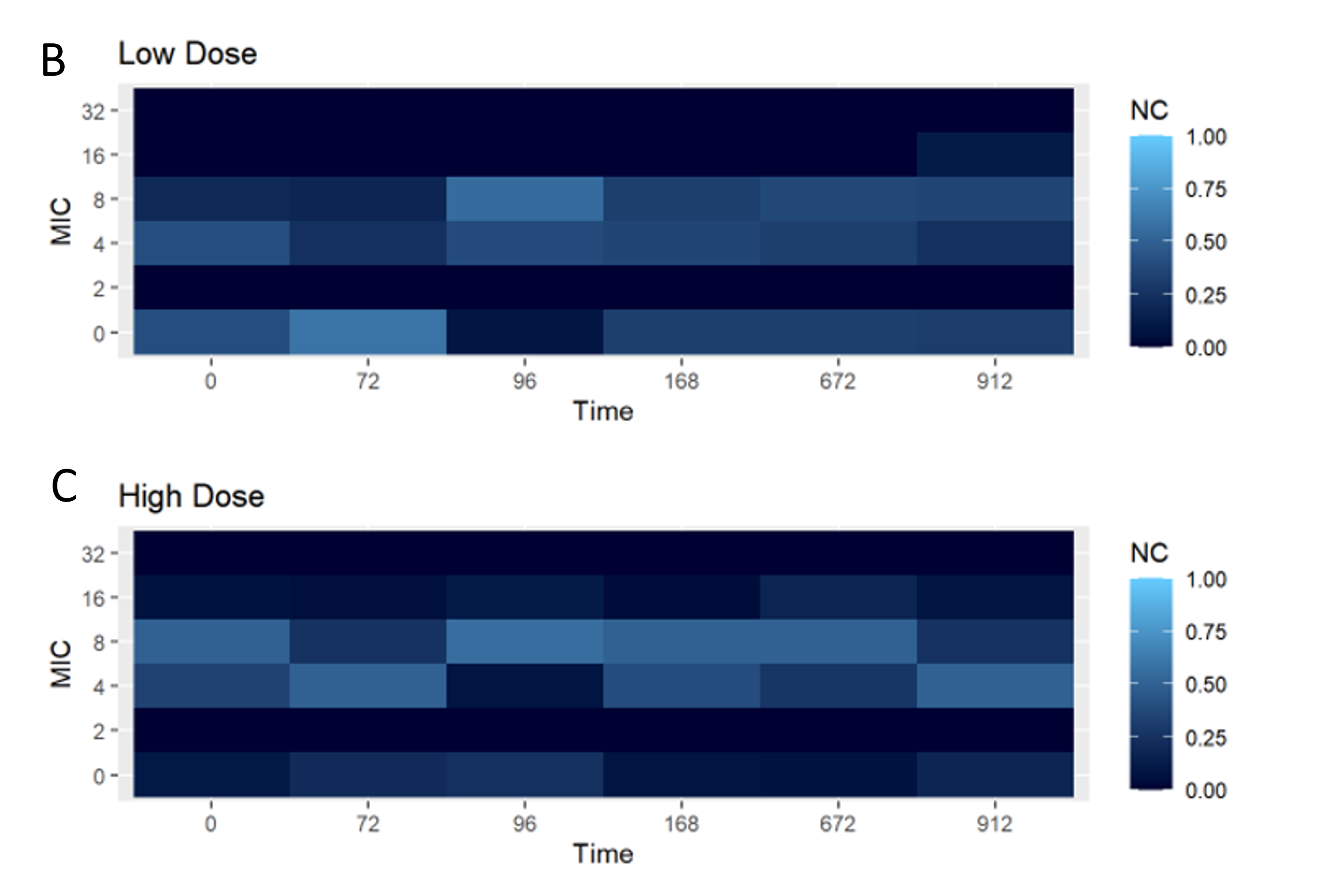

Supplement: Supplementary file 2 — Supplementary Figure S1. [file 41598_2024_55591_MOESM2_ESM.png]

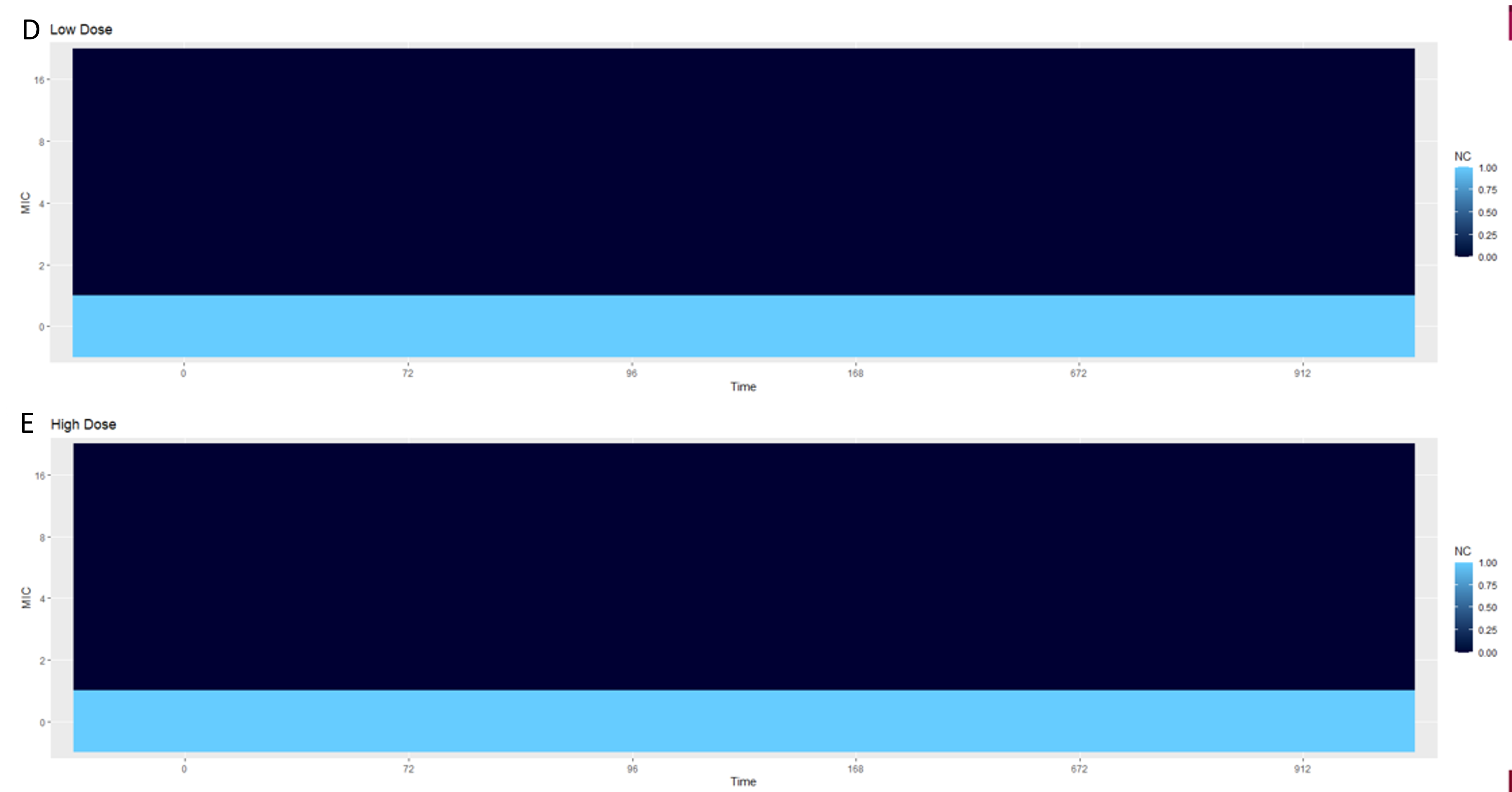

Supplement: Supplementary file 3 — Supplementary Figure S1. [file 41598_2024_55591_MOESM3_ESM.png]

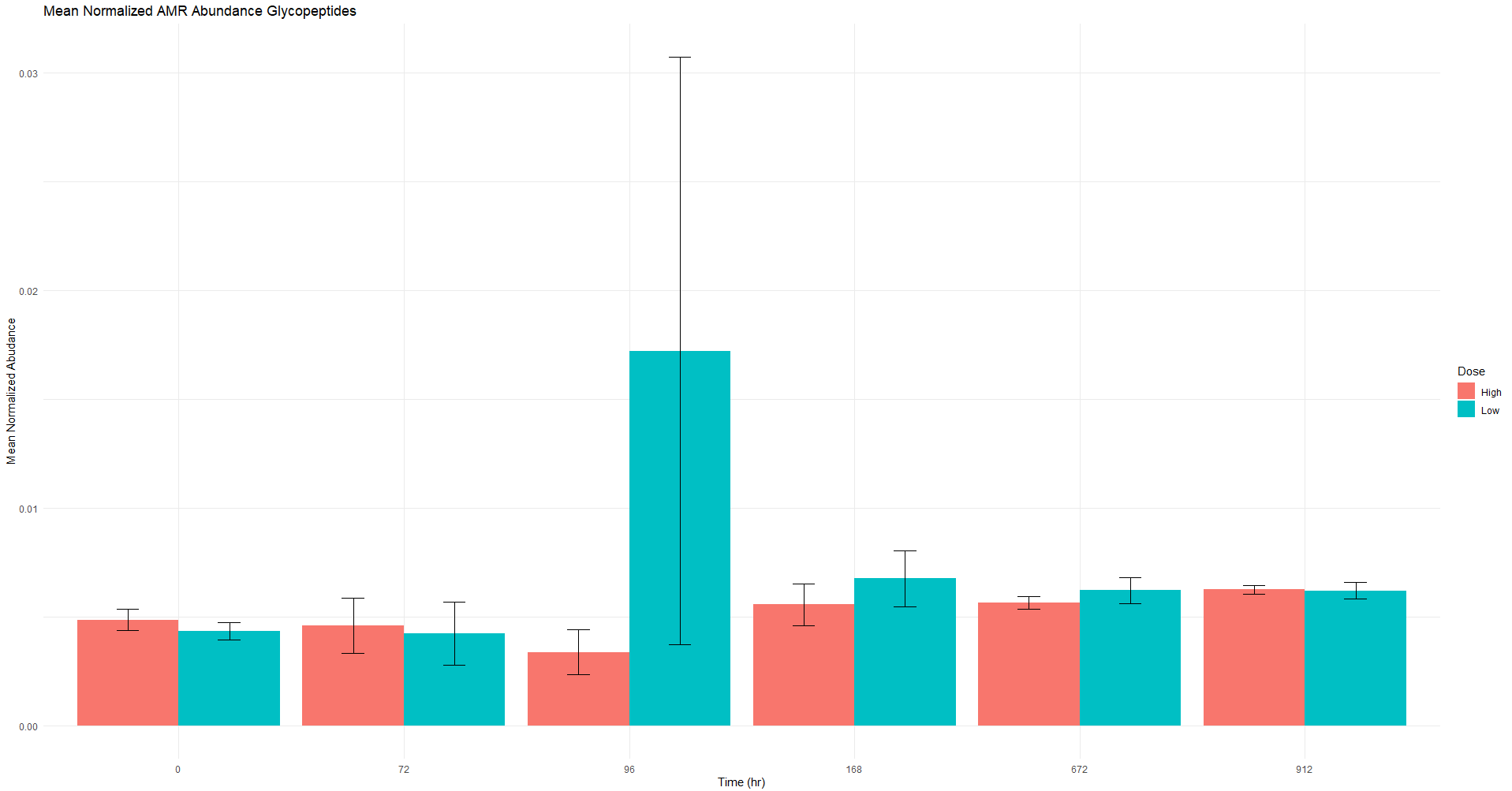

Supplement: Supplementary file 4 — Supplementary Figure S2. [file 41598_2024_55591_MOESM4_ESM.png]

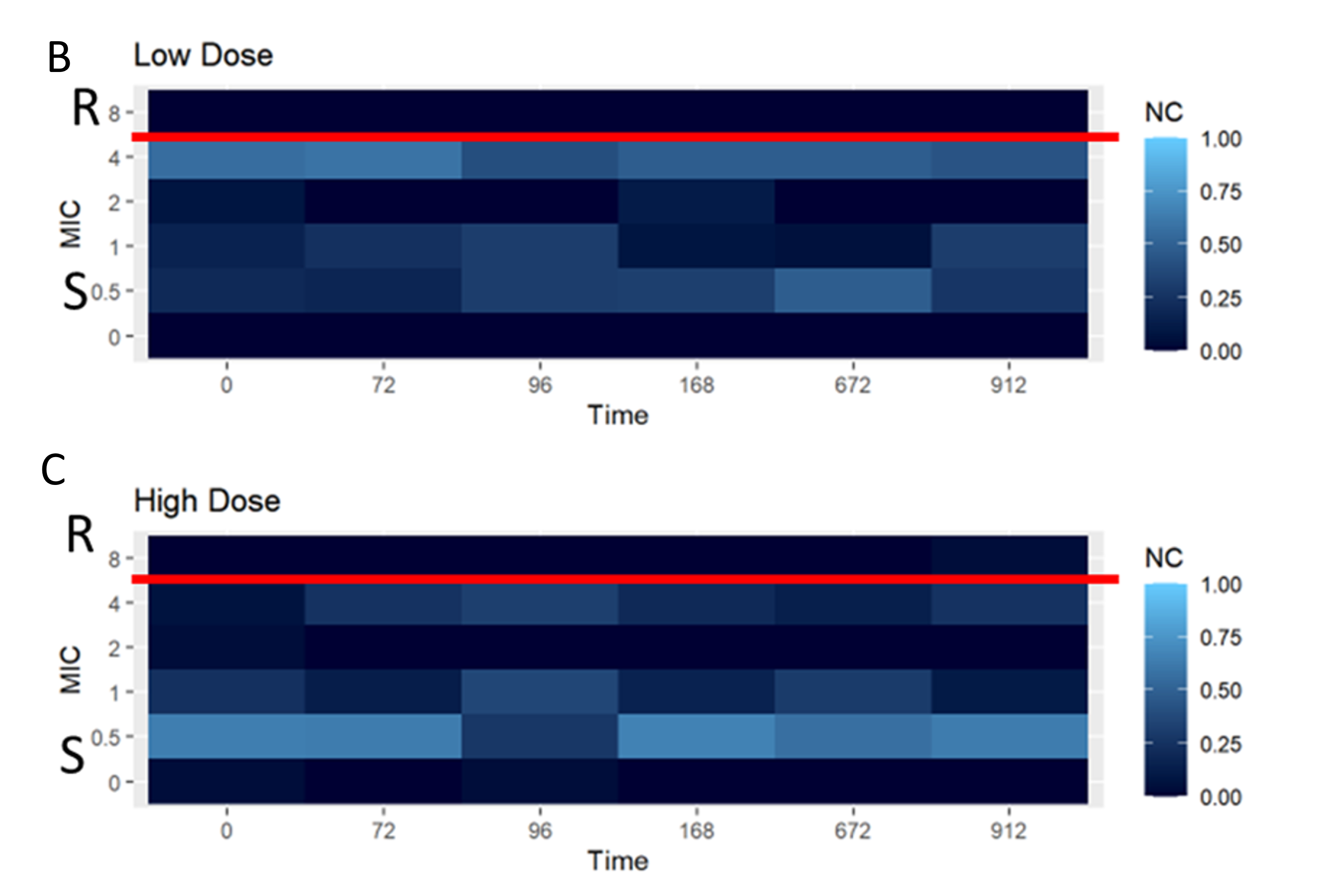

Supplement: Supplementary file 5 — Supplementary Figure S2. [file 41598_2024_55591_MOESM5_ESM.png]

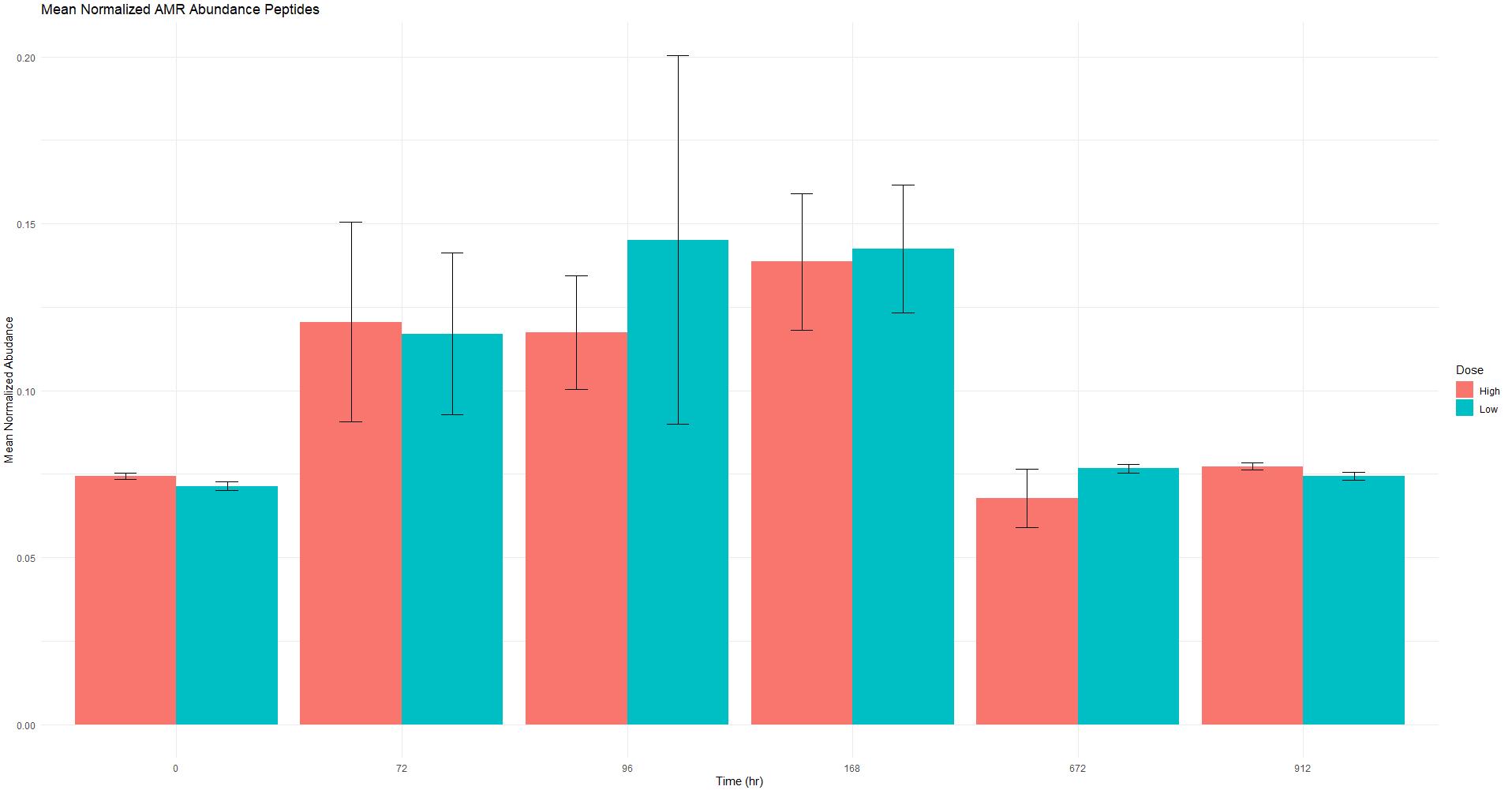

Supplement: Supplementary file 6 — Supplementary Figure S3. [file 41598_2024_55591_MOESM6_ESM.png]

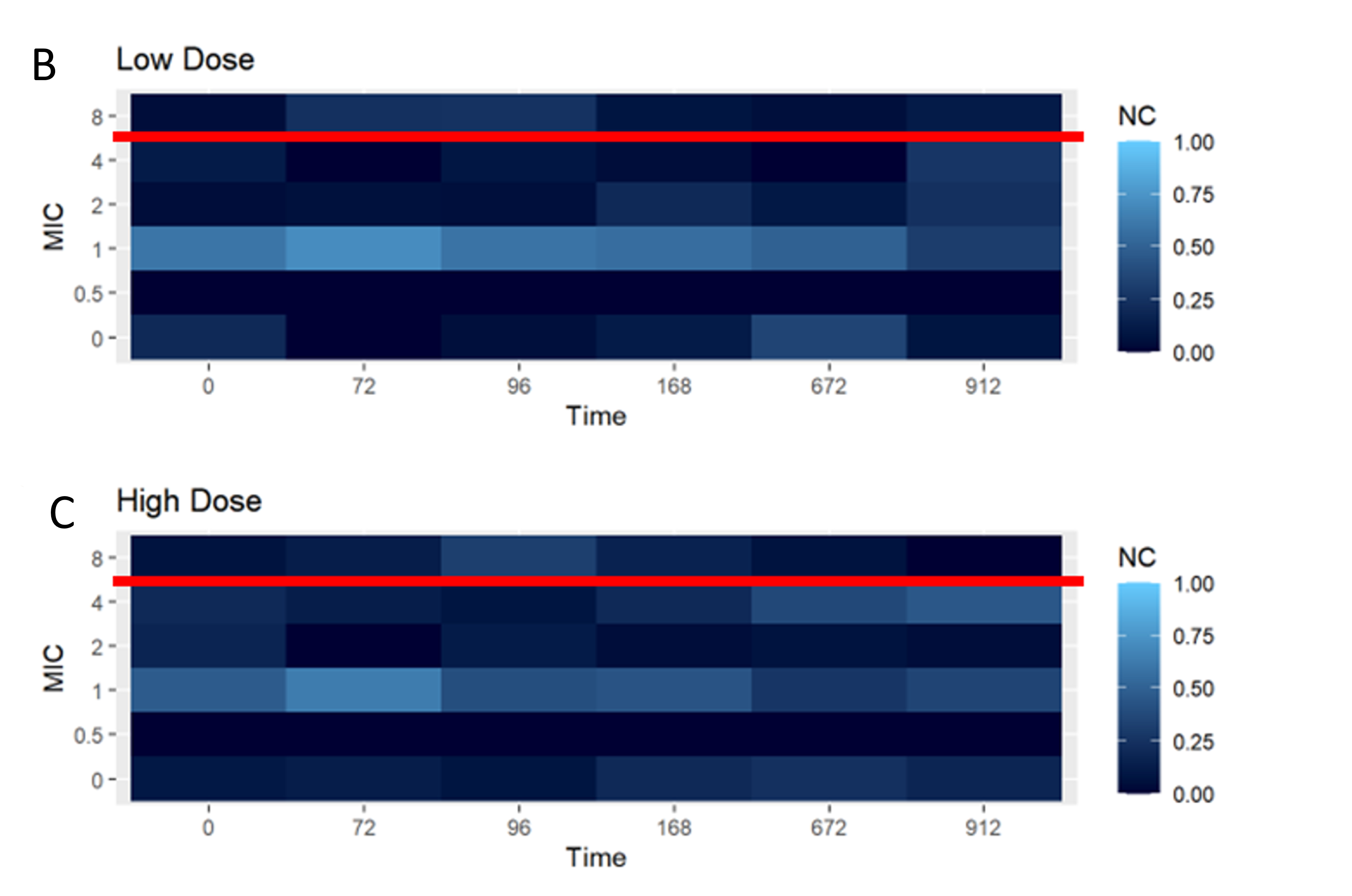

Supplement: Supplementary file 7 — Supplementary Figure S3. [file 41598_2024_55591_MOESM7_ESM.png]

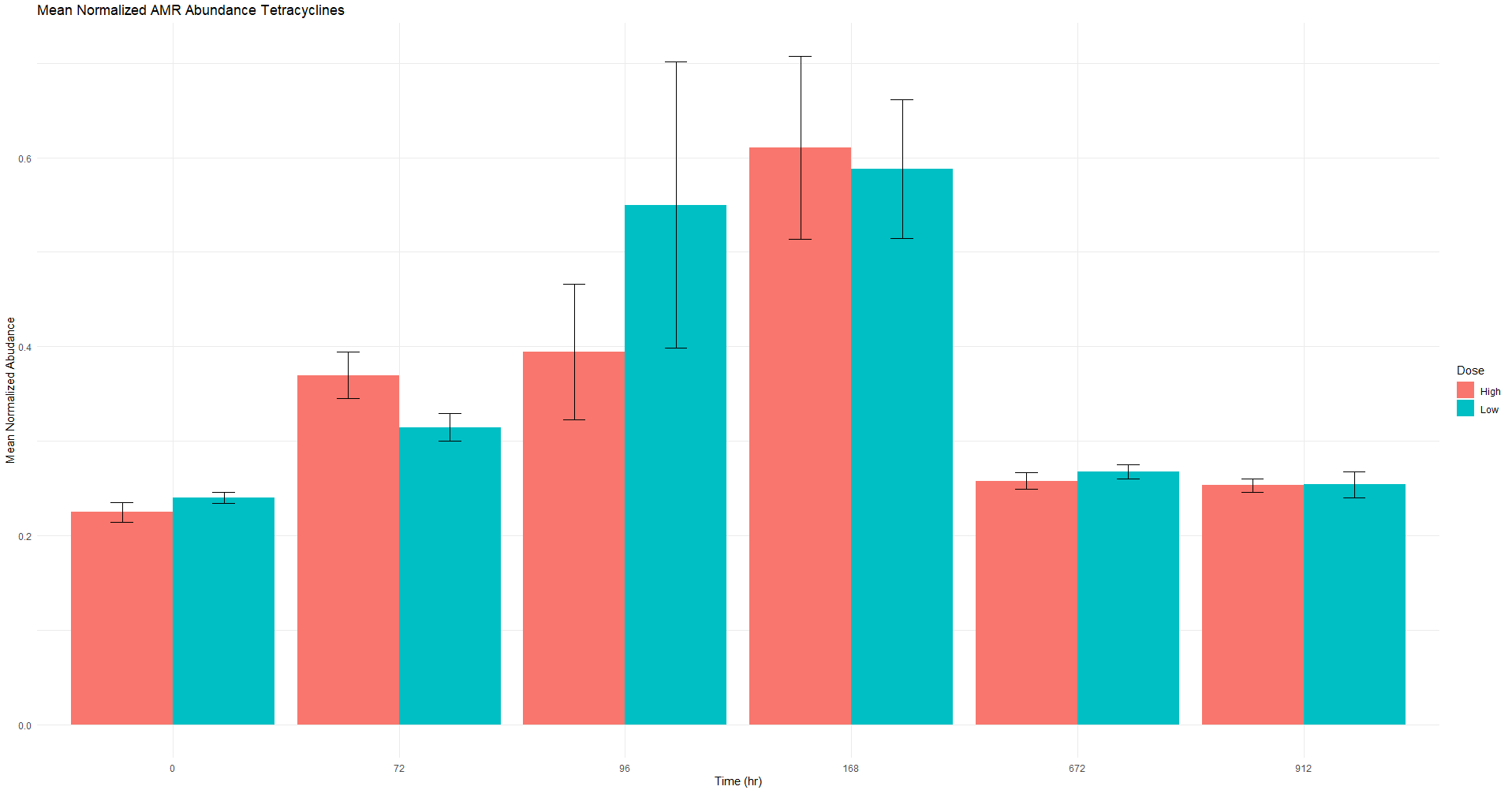

Supplement: Supplementary file 8 — Supplementary Figure S4. [file 41598_2024_55591_MOESM8_ESM.png]

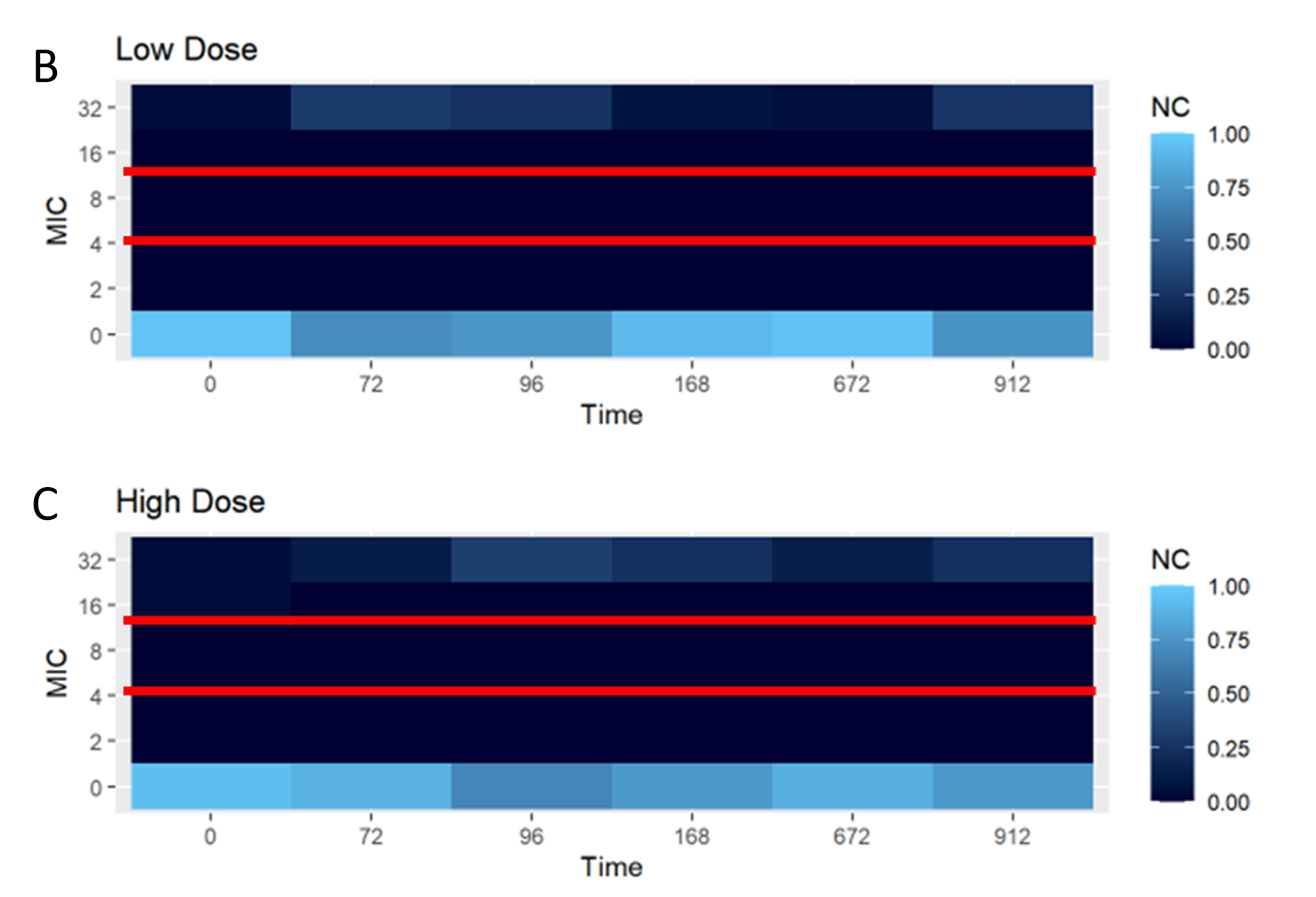

Supplement: Supplementary file 9 — Supplementary Figure S4. [file 41598_2024_55591_MOESM9_ESM.png]

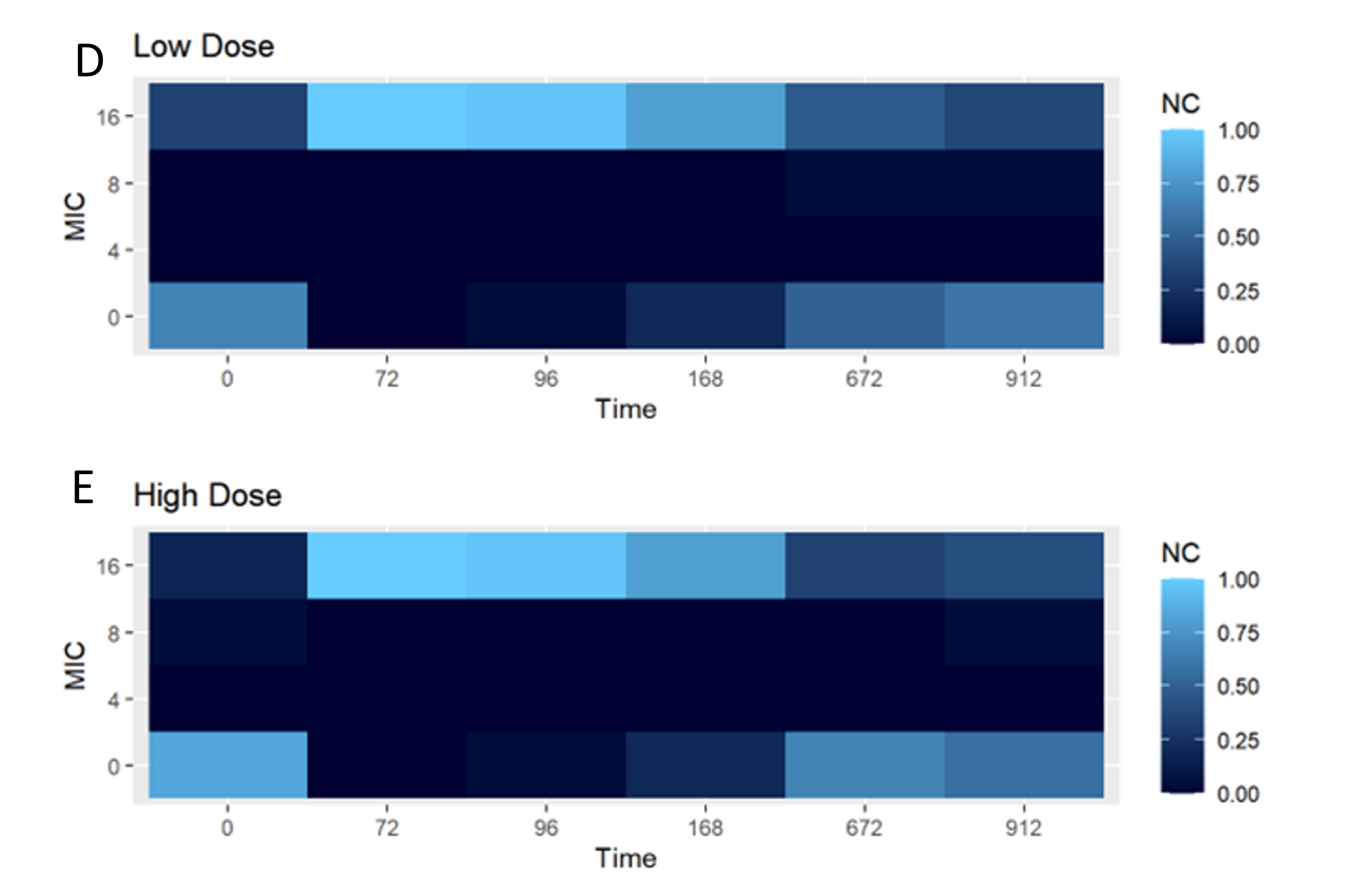

Supplement: Supplementary file 10 — Supplementary Figure S4. [file 41598_2024_55591_MOESM10_ESM.png]

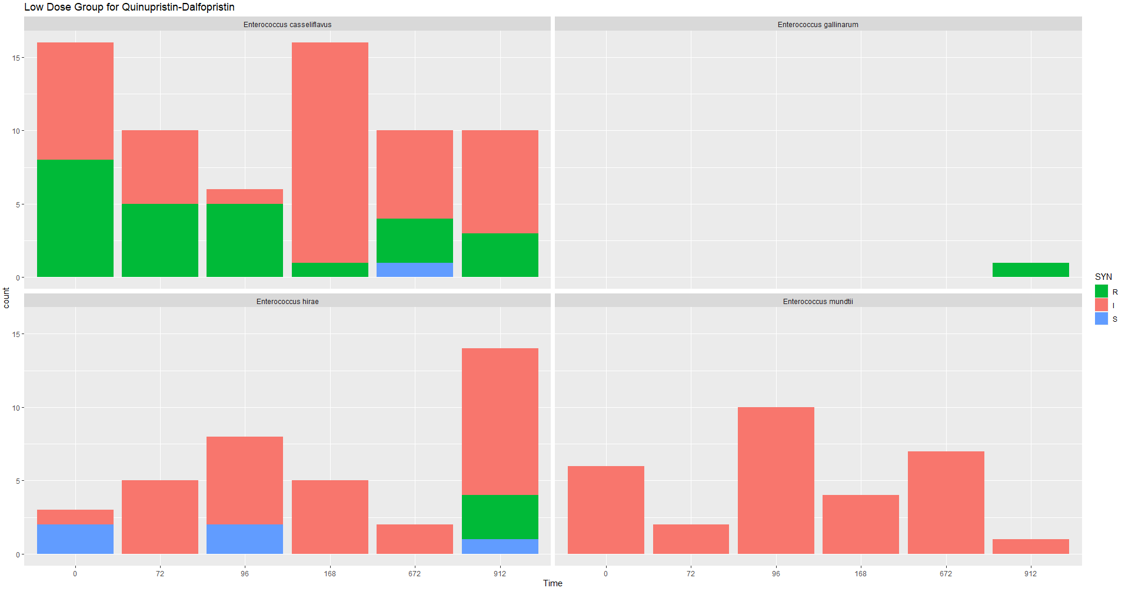

Supplement: Supplementary file 11 — Supplementary Figure S5. [file 41598_2024_55591_MOESM11_ESM.png]

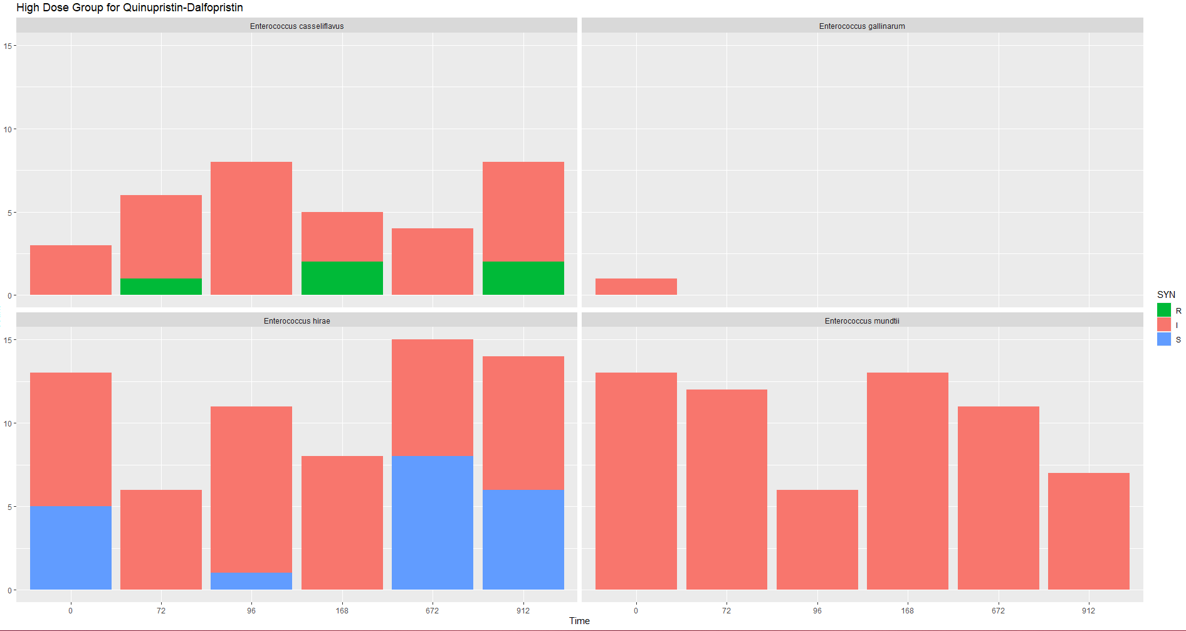

Supplement: Supplementary file 12 — Supplementary Figure S5. [file 41598_2024_55591_MOESM12_ESM.png]
